# A novel organic mineral complex prevented high fat diet-induced hyperglycemia, endotoxemia, liver injury and endothelial dysfunction in young male Sprague-Dawley rats

**DOI:** 10.1371/journal.pone.0221392

**Published:** 2019-08-26

**Authors:** Meli’sa S. Crawford, Eric Gumpricht, Karen L. Sweazea

**Affiliations:** 1 School of Life Sciences, Arizona State University, Tempe, Arizona, United States of America; 2 Isagenix International, LLC, Gilbert, Arizona, United States of America; 3 College of Health Solutions, Arizona State University, Phoenix, Arizona, United States of America; University of Melbourne, AUSTRALIA

## Abstract

The prevalence of metabolic syndrome (MetSyn) has risen 35% since 2012 and over two-thirds of Americans exhibit features characterizing this condition (obesity, dyslipidemia, hyperglycemia, insulin resistance and/or endothelial dysfunction). The aim of this study was to evaluate the effects of a novel dietary supplemental organic mineral complex (OMC) on these risk factors in a rodent model of MetSyn. Six-week old male Sprague-Dawley rats were fed either standard chow or a high-fat diet (HFD) composed of 60% kcal from fat for 10 weeks. Rats were also treated with OMC in their drinking water at either 0 mg/mL (control), 0.6 mg/mL, or 3.0 mg/mL. The HFD-treated rats exhibited significantly increased body mass (*p*<0.05), epididymal fat pad mass (*p*<0.001), waist circumference (*p* = 0.010), in addition to elevations in plasma endotoxins (*p*<0.001), ALT activity (*p*<0.001), fasting serum glucose (*p* = 0.025) and insulin concentrations (*p* = 0.009). OMC did not affect body weight or adiposity induced by the HFD. At the higher dose OMC significantly blunted HFD-induced hyperglycemia (*p* = 0.021), whereas both low and high doses of OMC prevented HFD-induced endotoxemia (*p* = 0.002 and <0.001, respectively) and hepatocyte injury (ALT activity, *p*<0.01). Despite evidence of oxidative stress (elevated urinary H_2_O_2_ *p* = 0.032) in HFD-fed rats, OMC exhibited no demonstrable antioxidative effect. Consistent with prior studies, mesenteric arteries from HFD rats had more uncoupled eNOS (*p* = 0.006) and iNOS protein expression *(p* = 0.027) in addition to impaired endothelium-dependent vasodilation that was abrogated by the high dose of OMC (*p*<0.05). This effect of OMC may be attributed to the high nitrate content of the supplement. These findings suggest that the OMC supplement, particularly at the higher dose, ameliorated several risk factors associated with MetSyn via a non-antioxidant-dependent mechanism.

## Introduction

Current estimates predict that approximately 20–25% of all US adults have metabolic syndrome (MetSyn), a constellation of symptoms characterized by abdominal obesity, dyslipidemia, hypertension, insulin resistance, and a pro-inflammatory or thrombotic state [1]. Several interrelated environmental and lifestyle factors contribute toward development of MetSyn and culminate in an increased risk for developing cardiovascular disease and type 2 diabetes [2]. The pathogenesis and underlying mechanisms responsible for developing MetSyn are still subject to intensive investigation; however, the role of excessive caloric intake and lack of physical activity are well established contributory factors. As a consequence of these factors, metabolic derangements including glucose and lipid dysregulation, insulin resistance, and endothelial dysfunction may be further promoted by oxidative stress and activation of inflammatory cascades [3, 4].

Because central obesity and visceral adiposity are two common hallmarks of MetSyn, management of the condition inevitably involves pharmacological and/or dietary-induced weight loss and weight loss maintenance [5], as well as increased physical activity [6]. Other dietary patterns recommended for MetSyn management include reductions in dietary sodium, simple carbohydrates, trans-fatty acids, cholesterol, and saturated fatty acids as exemplified by individuals adhering to the Mediterranean or Dietary Approaches to Stop Hypertension (DASH) diets [7, 8]. In addition to these overall comprehensive modifications to diet and lifestyle, several dietary components or bioactives have been reported to positively influence one or more MetSyn risk factors. Included here are turmeric, polyphenols, cinnamon, garlic, omega-3 fatty acids, and cruciferous vegetables [9, 10]. Ultimately, management and greater understanding of the underlying pathophysiology and biochemical mechanisms will result in greater potential therapeutic modalities in treating and preventing MetSyn.

As part of our longstanding interest in elucidating the effects of metabolic dysfunctions associated with vascular and endothelial derangements, we have utilized a high-fat diet (HFD)-induced rodent model of MetSyn. Previously we observed that rats fed HFD for 6 weeks exhibited hallmarks of MetSyn including weight gain, visceral adiposity, glucose and lipid dysregulation [11, 12]. Moreover, we previously demonstrated HFD impairs nitric oxide (NO)-dependent and independent vasodilation in isolated small resistance mesenteric arteries [11]. Interestingly, these effects were reversed by antioxidants or anti-inflammatory interventions [11] thereby providing potential avenues for MetSyn management.

The purpose of the current study was to characterize the effect of a novel and unique dietary supplemental organic mineral complex (OMC) on the pathophysiological and biochemical disturbances observed in this HFD-induced model of MetSyn. We observed this supplement, an organic mineral complex derived from plant and soil fractions, significantly attenuated several risk factors associated with MetSyn.

## Materials and methods

### Animal model

All procedures were approved by the Arizona State University Institutional Animal Care and Use Committee (17-1563R). Six-week old male Sprague-Dawley rats (157.5 ± 1.32 g body mass; n = 42) were purchased from Envigo (formerly Harlad Teklad) and randomly divided into two groups: either maintained on their routine standard chow maintenance diet (Teklad Global 2018, Indianapolis, IN) or switched to a 60% kcal from fat diet (Cat. No. D12492; Research Diets Inc, New Brunswick, NJ) as previously described [13]. Briefly, the composition of the chow diet in kcal was 24% protein, 58% carbohydrates and 18% fat whereas the high fat diet was comprised of similar total protein (20%), higher fat (60%) and lower carbohydrates (20%). The chow diet was mainly derived from plants (wheat, corn, soybean) whereas the HFD contained nutrients from several animal sources (lard, casein, lactic and 30 mesh), which models western diet intake. In addition, the carbohydrate content of the HFD was derived from both corn and added sucrose. Rats were fed the respective diets for 10 weeks.

Rats in each dietary group were administered 0 (control), 0.6, or 3.0 mg/mL OMC (provided by Isagenix International, LLC) in their drinking water throughout the diets. The dosage of OMC chosen for this study was based upon previous work by Deneau et al. [14] who evaluated a very similar ingredient in a mouse model of genetically-induced diabetes. Food and OMC-treated water were replaced every 2–3 days to prevent spoiling. Rats were exposed to 12:12 h light: dark cycle and were singly-housed to avoid coprophagic cross-contamination as the gut microbiome may contribute to systemic lipopolysaccharide (LPS) concentrations. Animals were allowed free access to water and food *ad libitum*. Study animals were euthanized by an overdose of sodium pentobarbital (200 mg/kg, i.p.) at the end of the 10 weeks.

### OMC supplement

Organic mineral complex (OMC) used in the current study is a proprietary nutritional ingredient marketed by the study sponsor (Isagenix International, LLC, Gilbert, AZ) and is a constituent of their trademarked ingredient “Ionic Alfalfa”. The ingredient is a complex natural product that is obtained in the initial raw state from several mineral mines in North America. It is extracted, isolated and manufactured for use as a dietary supplement by Mineral Biosciences, LLC (Goodyear, AZ) and is self-affirmed GRAS (“Generally Recognized as Safe”). OMC is an ancient plant and soil-derived material, similar to shilajit, that undergoes significant, proprietary isolation techniques to yield a soil-based blend containing over 50 minerals along with a high concentration of fulvic acid. Further chemical and biological characteristics are detailed in Table 1.

**Table 1:** Physical, chemical, and functional characteristics of OMC.

Component Measured	Concentration or Value	Analytical Methodology or Source
Total Minerals	142391 ppm	ICP
Calcium	49610 ppm	ICP
Sulfur	28040 ppm	ICP
Potassium	15420 ppm	ICP
Sodium	14990 ppm	ICP
Magnesium	12630 ppm	ICP
Nitrate (NO_3_^-^)	1230 ppm	Univ. Wisconsin Soil and Forage Analysis Laboratory
Fulvic Acids	14.9%	Lamar et al., 2014 [40]
Humic Acids	<0.1%	Lamar et al., 2014 [40]
Protein	23 mg/g	CLG-PRO4 determination by combustion
Nucleic Acids	ND	DAPI (4’,6-Diamidino-2-phenylindole)-staining
Total Polyphenols	0.24%	Folin-Ciocalteu
ORAC Score-Hydrophilic	24.92	Brunswick Laboratories
ORAC Score-Hydrophobic	5.44	Brunswick Laboratories

### Morphometrics

Body mass was measured weekly to assess changes in response to the diet and OMC treatments. Nasoanal length, tail length, and abdominal circumference (immediately anterior to the hindleg) were measured using a flexible tape measure at the end of the 10-week trial. Following euthanasia, blood was collected by cardiac puncture and the plasma isolated and frozen at -80°C until use. The epididymal fat pad was extracted from each animal to assess adiposity as previously described [11]. Lee’s Index of Obesity was calculated as cube root of body mass (g) / nasoanal length (cm)) x 1000 [15, 16].

### Glucoregulatory variables

Rats were food-restricted by providing an aliquot of food (2g/rat at baseline and 4g/rat at weeks 6 and 10) at 6:00 pm the night prior to the fasting blood draws. The following morning fasting blood samples (300–500 μL) were collected from the tail vein at baseline, weeks 6, and 10. Serum was then separated from whole blood and stored at -80°C until analyses. Fasting serum glucose concentrations were measured via the glucose oxidase method using a commercially available kit (Cat. No. 10009582, Cayman Chemical, Ann Arbor, MI) according to the manufacturer’s protocol. Fasting serum insulin concentrations were measured using a commercially available kit (Cat. No. 90060, Crystal Chem, Elk Grove Village, IL). Quantitative Insulin Sensitivity Check Index (QUICKI) was calculated as (1/(log insulin (mU/L) + log glucose (mM/L)). QUICKI is a validated surrogate measure of insulin sensitivity used in both clinical and animal studies [17, 18]. Studies of rats have shown that QUICKI is a better indicator of insulin sensitivity than HOMA-IR as it does not include a human specific-normalizing factor [17].

### Biomarkers of oxidative stress and hepatocyte injury

Plasma superoxide dismutase (SOD) activity was measured at the end of the study using commercially available kits (Cat. No. 706002, Cayman Chemical, Ann Arbor, Michigan). Urine hydrogen peroxide and creatinine concentrations were measured using commercially available kits (Cat. No. ab102500, Abcam, Cambridge, MA; Cat. No. CR01, Oxford Biochemical Research, Rochester Hills, MI). Activity of plasma ALT and AST were also measured at the end of the 10-week feeding protocol using commercially available kits (Cat. No. MAK052 and MAK055, respectively; Sigma Aldrich, St. Louis, MO).

### Quantification of plasma endotoxins

Plasma lipopolysaccharide concentrations were quantified with a commercially available kit (Cat. No. 88282, Thermo Fisher Scientific Rockford, IL) per the manufacturer’s protocol.

### Endothelium-dependent vasodilation

Stock solutions of acetylcholine (ACh, 1.0 M, Sigma Aldrich) and phenylephrine (PE, 1.0 M, Sigma Aldrich) were prepared in deionized water, aliquoted, and stored at -20°C until use. Following euthanasia, a midline laparotomy was performed to remove the mesenteric arcade, which was immediately transferred to ice-cold HEPES-buffered saline (in mM: 134.4 NaCl, 6 KCl, 1 MgCl_2_, 1.8 CaCl_2_, 10 HEPES, and 10 glucose, pH 7.4 with NaOH). The arcade was pinned out in a silastic-coated dissection dish and mesenteric resistance arteries (~1mm length; 126 ± 3 μm, i.d.) were isolated and transferred to a vessel chamber (Cat. No. CH-1, Living Systems Instrumentation, St. Albans, VT) containing HEPES-buffered saline. Isolated arteries were then cannulated with glass pipettes, secured with silk ligatures, and stretched longitudinally to approximate *in situ* length. Vessels were then pressurized to 60 mmHg using a servo-controlled peristaltic pump (Living Systems Instrumentation) and the vessel chamber transferred to the stage of an inverted Nikon microscope for analysis. Vessels were continuously superfused with warm (37°C) physiological saline solution (PSS, in mM: 129.8 NaCl, 5.4 KCl, 0.5 NaH_2_PO_4_, 0.83 MgSO_4_, 19 NaHCO_3_, 1.8 CaCl_2_, and 5.5 glucose) at a rate of 10 mL/min. PSS was aerated with a gas mixture containing 21% O_2_, 6% CO_2_, balance N_2_ to maintain pH and oxygenation.

Following a 30-minute equilibration of isolated arteries in PSS, vessels were pre-constricted with increasing concentrations of PE in the superfusate until they reached 50% of their resting inner diameter. Endothelium-dependent vasodilation was assessed by exposing pre-constricted arteries to stepwise increases of the endothelium-dependent vasodilator ACh (10^−9^ to 10^−5^ M, 3 min per step) in the superfusate followed by a calcium-free PSS solution (in mM: 129.8 NaCl, 5.4 KCl, 0.5 NaH_2_PO_4_, 19.0 NaHCO_3_, 5.5 glucose, and 3 EDTA) to measure the passive inner diameter. Intraluminal diameter (i.d.) was continuously monitored from video microscopy of bright field images using an edge-detection Vessel Diameter System (IonOptix, Milton, MA, USA). Vasodilation was calculated as the percent reversal of PE-mediated vasoconstriction.

### Western blot analyses

Mesenteric arteries were isolated and snap-frozen on dry ice. Frozen arteries were homogenized in ice-cold tissue protein extraction reagent (T-PER, Cat. 78510, Thermo Fisher Scientific, Waltham, MA) containing HALT Protease Phosphatase Inhibitor Cocktail (Cat. 78446, Thermo Fisher Scientific) in 2 mL microcentrifuge tubes containing 1.5 mm zirconium beads using a BeadBug homogenizer (Benchmark Scientific, Edison, NJ). Homogenates were centrifuged at 14,000 rpm for 10 min at 4°C to remove insoluble debris and concentration of proteins in the supernatant was analyzed using the Bradford method (Bio-Rad, Hercules, CA). Tissue sample proteins (50 μg/lane) were resolved by 7.5% Tris-HCl sodium dodecyl sulfate polyacrylamide gel electrophoresis (SDS-PAGE) (Bio-Rad, Hercules, CA). Separated proteins were transferred to Immuno-Blot polyvinylidene difluoride (PVDF) membranes (Bio-Rad, Hercules, CA) and blocked overnight at 4°C in blocking buffer (100 ml Tween/Tris-buffered saline (TTBS), 3% BSA, 5% nonfat dry milk). For eNOS protein detection, membranes were washed in TTBS and incubated overnight at 4°C with mouse monoclonal antibody specific for eNOS (1:2500; Cat. 610296; BD Transduction Laboratories, San Jose, CA). For iNOS protein detection, membranes were incubated overnight at 4°C followed by a 4 hour incubation at room temperature with a mouse monoclonal antibody specific for iNOS (1:1000; Cat. 610431, BD Transduction Laboratories). Both membranes were incubated overnight with a rabbit polyclonal antibody to β-actin as a loading control (1:10,000; Cat. Ab8227; AbCam, Cambridge, MA). Membranes were then washed in TTBS and incubated with anti-mouse (1:5000 for eNOS, 1:2000 for iNOS) and anti-rabbit (1:5000) horseradish peroxidase-conjugated secondary antibodies (Cat. PI-2000 and PI-1000; Vector Laboratories, Burlingame, CA) for 1 hr at room temperature followed by washes in Tris-buffered saline (TBS) and a 1 min exposure to Pierce enhanced chemiluminescence western blotting substrate (Thermo Scientific, Rockford, IL). Immunoreactive bands were visualized by exposure to x-ray film (Kodak X-OMAT, Thermo Fisher Scientific, Pittsburgh, PA). The developed films were analyzed using ImageJ software (NIH) and eNOS as well as iNOS protein levels were normalized to β-actin and expressed as a ratio of the Chow control.

### Statistical analyses

All data are expressed as means ± SEM. Data collected at multiple time points (body mass, glucose, insulin, and endothelium-dependent vasodilation) were analyzed by two-way repeated measures ANOVA with diet and OMC dose as factors. Percent data were arcsine transformed to approximate normal distribution prior to analyses. All other data were analyzed by two-way ANOVA with diet and OMC dose as factors. Where significant effects were observed, Tukey posthoc analyses were used. A probability of ≤ 0.05 was accepted as statistically significant.

## Results

### Morphometrics

**Fig 1:**
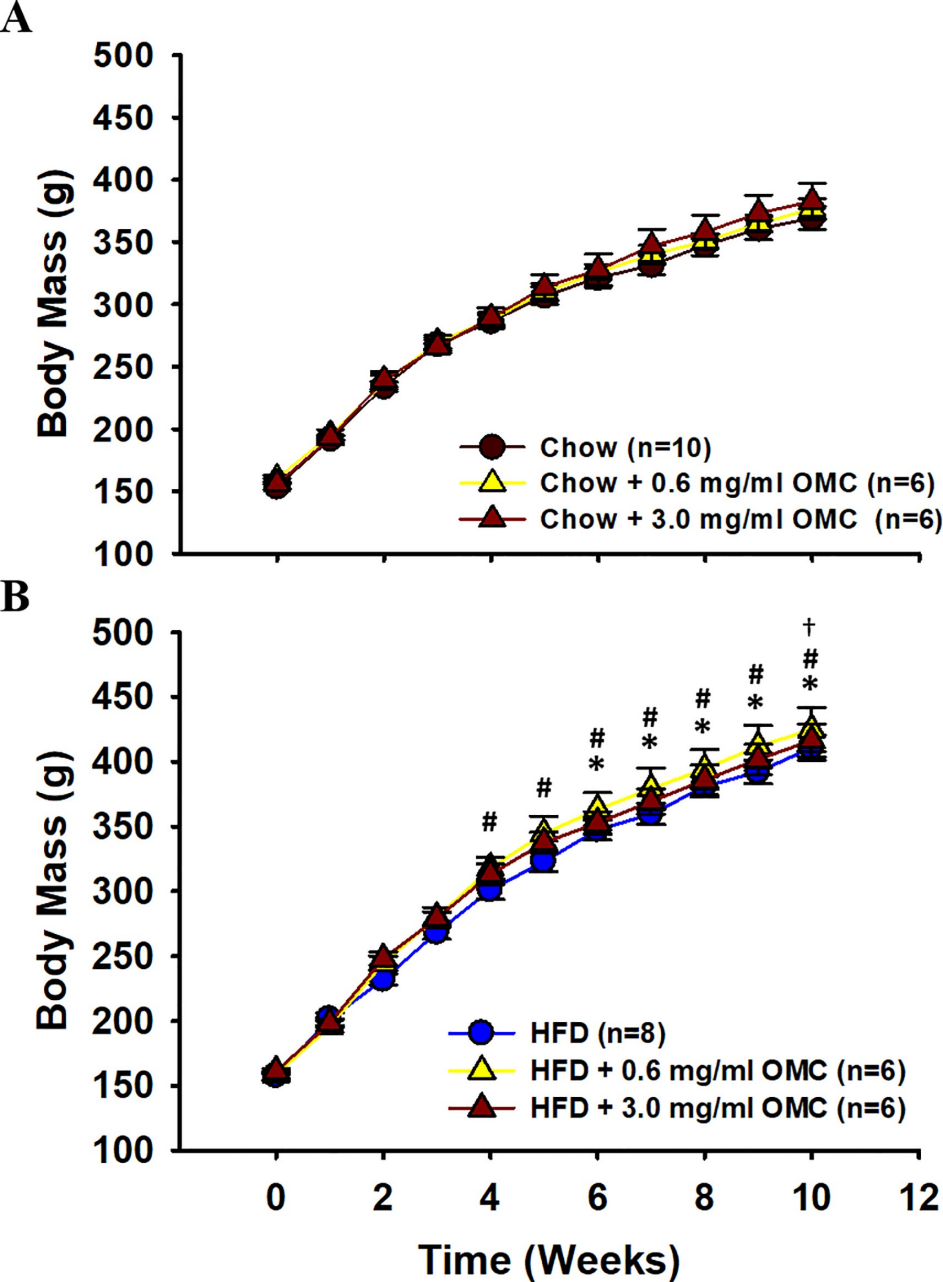
Effects of OMC treatment on body mass. Rats were fed either (A) standard rodent chow or (B) a 60% kcal from fat high fat diet (HFD) for 10 weeks. Data are expressed as mean ± SEM. Data were analyzed by two-way RM ANOVA (SigmaStat 3.0, Systat Software, San Jose, CA). *p<0.05 HFD vs Chow; **#**p<0.05 HFD + 0.6 mg/ml OMC vs Chow + 0.6 mg/ml OMC, †p<0.05 HFD + 3 mg/ml OMC vs Chow + 3 mg/ml OMC; n = 6–10 per group.

HFD rats gained significantly more weight compared to respective Chow-fed controls (Fig 1). Similarly, epididymal fat pad mass, waist circumference, and naso-anal length were all significantly increased in HFD rats compared to controls, although tail length was not different between groups (Table 2). Lee’s Index of Obesity was not significantly different between HFD and Chow-fed rats indicating that the rats were simply overweight as opposed to obese (Table 2). OMC did not affect any morphometric variable (Fig 1, Table 2).

**Table 2:** Morphometrics at ten weeks.

	Control	0.6 mg/ml OMC	3.0 mg/ml OMC
**Epididymal fat pad mass (g)**^a^			
Chow	3.70 ± 0.16 (10)	4.14 **±** 0.24 (6)	3.89 ± 0.41 (6)
HFD	**6.09 ± 0.32 (8)#**	**7.44 ± 1.09 (6)#**	**6.67 ± 0.65 (6)#**
**Waist circumference (cm)**			
Chow	16.8 ± 0.28 (10)	17.5 ± 0.17 (6)	17.3 ± 0.20 (6)
HFD	**17.9 ± 0.25 (8)#**	**18.9 ± 0.62 (6)#**	**18.3 ± 0.24 (6)#**
**Tail length (cm)**			
Chow	21.3 ± 0.27 (10)	21.1 ± 0.29 (6)	21.4 ± 0.51 (6)
HFD	21.3 ± 0.22 (8)	21.5 ± 0.26 (6)	21.5 ± 0.31 (6)
**Naso-anal length (cm)**			
Chow	22.3 ± 0.15 (10)	22.1 ± 0.14 (6)	22.5 ± 0.24 (6)
HFD	**23.1 ± 0.14 (8)#**	**23.1 ± 0.20 (6)#**	**23.2 ± 0.30 (6)#**
**Lee’s Index of Obesity**			
Chow	321.1 ± 1.7 (10)	327.3 ± 3.3 (6)	322.9 ± 2.5 (6)
HFD	321.9 ± 1.7 (8)	324.7 ± 2.8 (6)	322.0 ± 1.8 (6)

Data expressed as mean ± SEM (n). Data analyzed by two-way ANOVA with diet and OMC dose as factors.

#*p*<0.02 vs respective Chow treated animal.

^a^Data was log transformed prior to statistical analyses to approximate normality. Lee’s Index of Obesity = (cube root of body mass (g) / nasoanal length (cm)) x 1000 [15, 16].

### Glucoregulatory variables

Rats developed significant hyperglycemia following 6 and 10 weeks of HFD (*p*<0.001 and *p* = 0.025, respectively) compared to Chow controls (Table 3). While administration of low dose OMC (0.6 mg/mL) tended to reduce HFD-induced hyperglycemia (*p* = 0.067), high dose OMC (3 mg/mL) significantly prevented hyperglycemia at 10 weeks (*p* = 0.021). OMC had no effect on fasting serum glucose concentrations in Chow-fed animals. By 10 weeks differences in fasting serum insulin were significant between Chow and HFD-fed rats (*p* = 0.009). Similarly, fasting serum insulin concentrations tended to be higher after 6 weeks in HFD rats treated with 0.6 mg/mL OMC compared to the respective Chow controls (*p* = 0.064). QUICKI was lower in HFD control rats after 6 and 10 weeks compared to Chow controls (*p*<0.001). HFD rats supplemented with 0.6 mg/mL and 3.0 mg/mL OMC also had lower QUICKI than Chow animals at week 6 (*p* = 0.031 and 0.008, respectively) but not at week 10 (*p* = 0.145 and 0.552, respectively). OMC treatment did not significantly affect QUICKI in Chow-fed animals nor did it significantly alter QUICKI among animals fed HFD.

**Table 3:** Fasting biochemical parameters.

	Week 6		Week 10	
	Mean ± SEM (n)	Range	Mean ± SEM (n)	Range
**Serum glucose (mM/L)**				
Chow	6.88 ± 0.28 (10)	[5.33–8.28]	7.08 ± 0.17 (10)	[5.82–7.71]
Chow + 0.6 mg/ml OMC	7.43 ± 0.36 (6)	[6.60–9.09]	7.34 ± 0.30 (6)	[6.22–8.35]
Chow + 3 mg/ml OMC	6.73 ± 0.45 (6)	[5.13–8.04]	7.01 ± 0.17 (6)	[6.52–7.61]
HFD	8.83 ± 0.41 (8)*	[7.74–10.8]	8.32 ± 0.33 (8)*	[7.09–9.98]
HFD + 0.6 mg/ml OMC	8.01 ± 0.34 (6)	[7.36–9.63]	7.30 ± 0.04 (6)	[7.17–7.43]
HFD + 3 mg/ml OMC	7.91 ± 0.29 (6)	[7.14–9.15]	**7.07 ± 0.19 (6)#**	[6.67–7.88]
**Serum insulin (mU/L)**				
Chow	12.9 ± 2.73 (10)	[7.05–36.3]	12.2 ± 1.27 (10)	[8.89–22.7]
Chow + 0.6 mg/ml OMC	14.7 ± 3.28 (6)	[9.92–30.3]	16.3 ± 2.99 (6)	[9.50–27.9]
Chow + 3 mg/ml OMC	12.4 ± 2.11 (6)	[8.07–20.5]	15.2 ± 1.69 (6)	[10.0–20.4]
HFD	29.2 ± 5.52 (8)	[10.9–51.3]	32.8 ± 3.80 (8)*	[21.4–52.5]
HFD + 0.6 mg/ml OMC	34.6 ± 7.41 (6)	[15.6–64.1]	34.3 ± 6.55 (6)	[15.4–55.6]
HFD + 3 mg/ml OMC	30.3 ± 9.18 (6)	[15.5–75.0]	25.8 ± 5.36 (6)	[15.6–49.0]
**QUICKI**				
Chow	0.54 ± 0.02 (10)	[0.42–0.64]	0.52 ± 0.01 (10)	[0.45–0.58]
Chow + 0.6 mg/ml OMC	0.51 ± 0.02 (6)	[0.41–0.55]	0.49 ± 0.02 (6)	[0.42–0.57]
Chow + 3 mg/ml OMC	0.54 ± 0.03 (6)	[0.45–0.62]	0.50 ± 0.01 (6)	[0.47–0.54]
HFD	0.43 ± 0.02 (8)*	[0.37–0.52]	0.42 ± 0.01 (8)*	[0.38–0.46]
HFD + 0.6 mg/ml OMC	0.42 ± 0.02 (6)*	[0.36–0.49]	0.43 ± 0.02 (6)	[0.38–0.49]
HFD + 3 mg/ml OMC	0.44 ± 0.02 (6)*	[0.36–0.48]	0.45± 0.02 (6)	[0.40–0.49]

Data expressed as mean ± SEM (n). Data analyzed by two-way RM ANOVA.

#p<0.05 vs HFD control at the same time point

*p<0.05 vs respective Chow at the same time point.

### Biomarkers of oxidative stress

**Fig 2:**
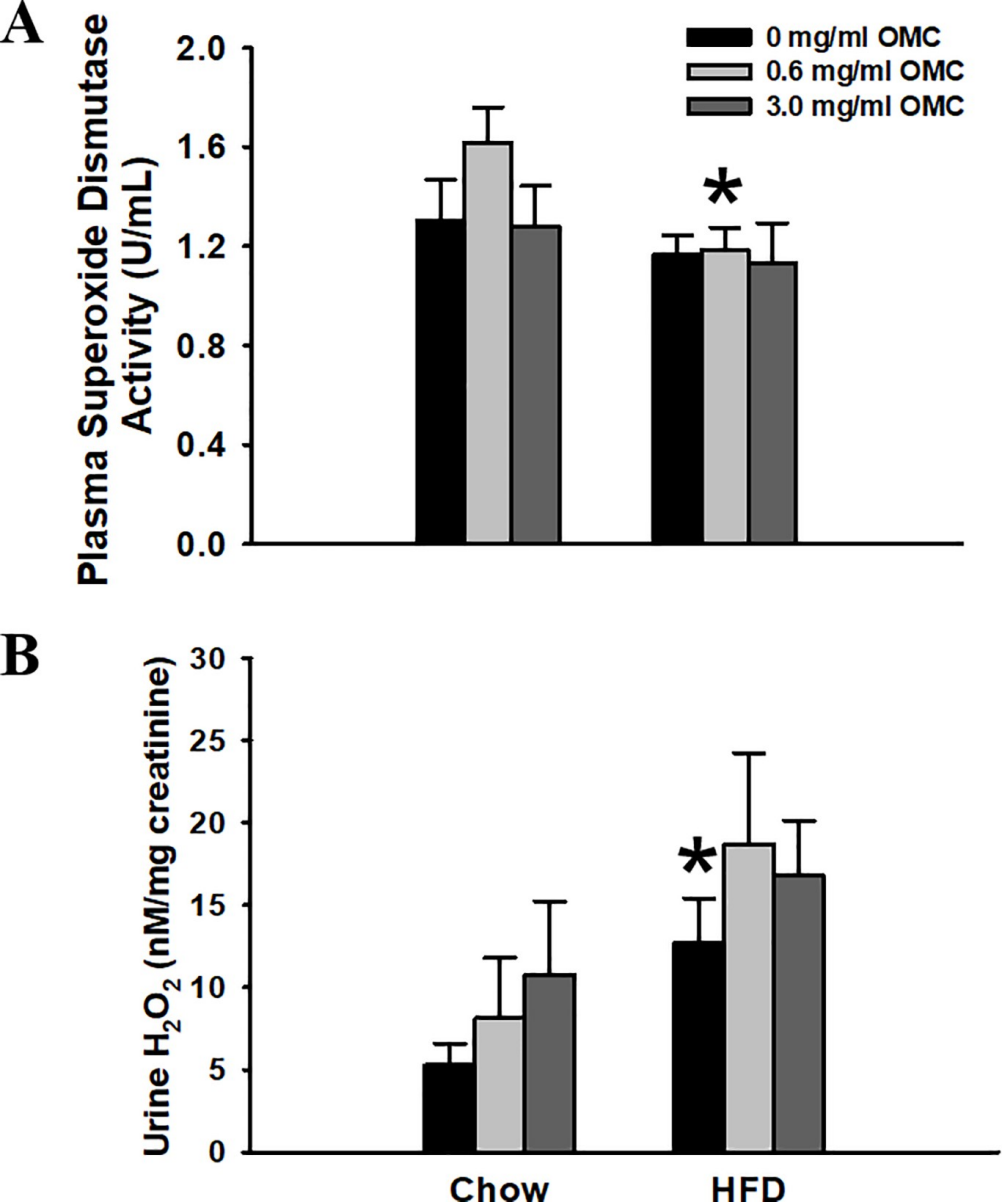
Plasma superoxide dismutase and urinary hydrogen peroxide concentrations. (A) Plasma superoxide dismutase (SOD) activity and (B) urine hydrogen peroxide (H_2_O_2_) concentrations normalized to urine creatinine. Data are expressed as mean ±SEM and analyzed by two-way ANOVA, *n* = 6–10 per group for plasma and *n* = 4–8 per group for urine. *p<0.05 HFD vs Chow animals receiving the same dose of OMC.

Plasma SOD activity was significantly higher in Chow rats treated with 0.6 mg/mL OMC compared to HFD-fed rats treated with the same dose (*p* = 0.047; Fig 2A). This difference was likely driven by the elevation in SOD activity in the Chow animals. No other changes in SOD activity were observed. HFD-fed rats without OMC treatment had significantly elevated urinary concentrations of H_2_O_2_ compared to Chow-fed untreated animals (*p* = 0.032). Urinary H_2_O_2_ concentrations were not affected by OMC (Fig 2B).

### Quantification of plasma endotoxins

**Fig 3:**
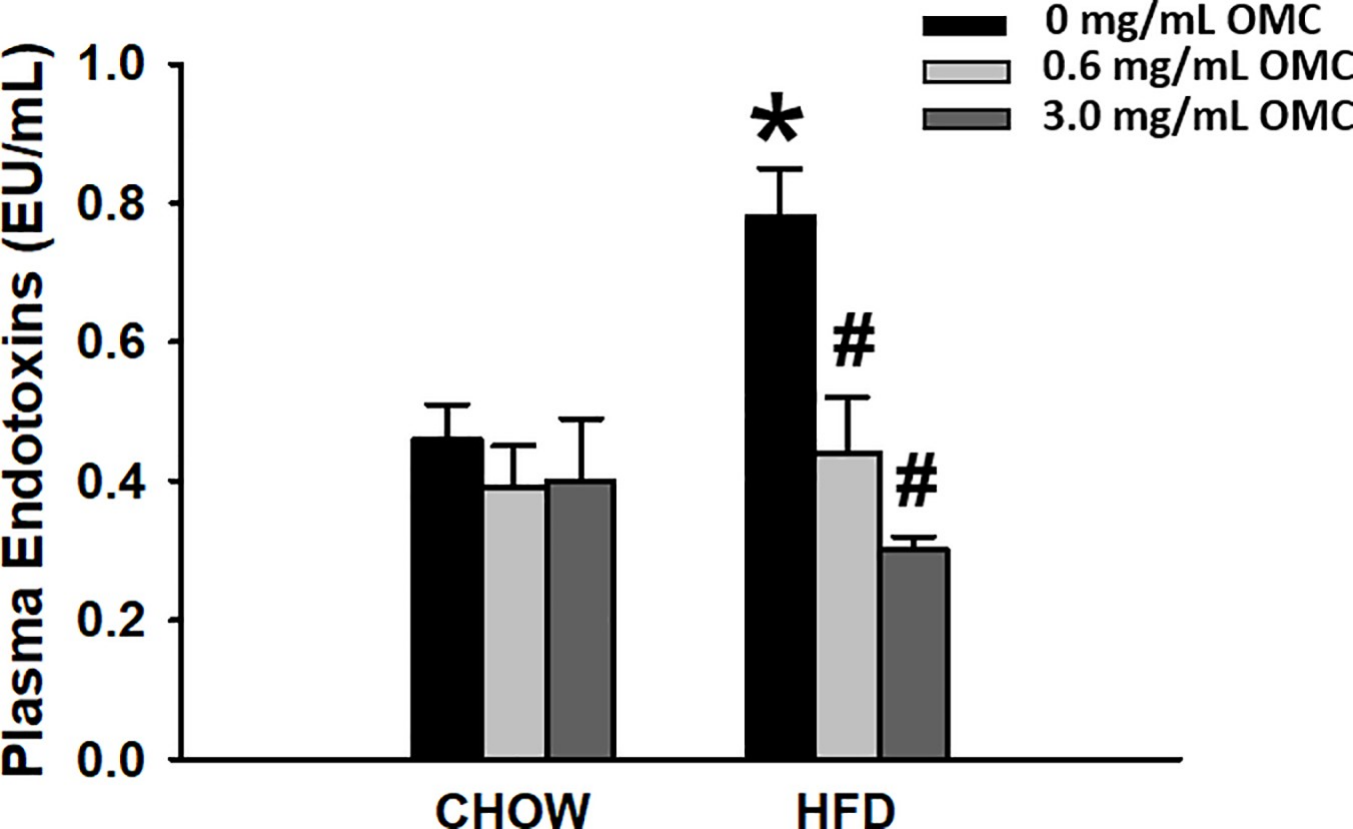
Plasma lipopolysaccharide (LPS) concentrations. Data are expressed as mean ±SEM and analyzed by Two-way ANOVA, *n* = 6–10 per group. *p<0.05 HFD vs Chow; **#**p<0.05 HFD + 0.6 mg/ml OMC vs Chow + 0.6 mg/ml OMC.

Plasma endotoxins were significantly elevated in the HFD rats compared to Chow control (*p*<0.05; Fig 3). However, treatment with OMC significantly blunted HFD-induced endotoxemia at both 0.6 and 3.0 mg/mL OMC (*p*<0.05; Fig 2C). No effect of OMC toward plasma endotoxins were observed in Chow control-fed rats.

### Biomarkers of hepatocyte injury

**Fig 4:**
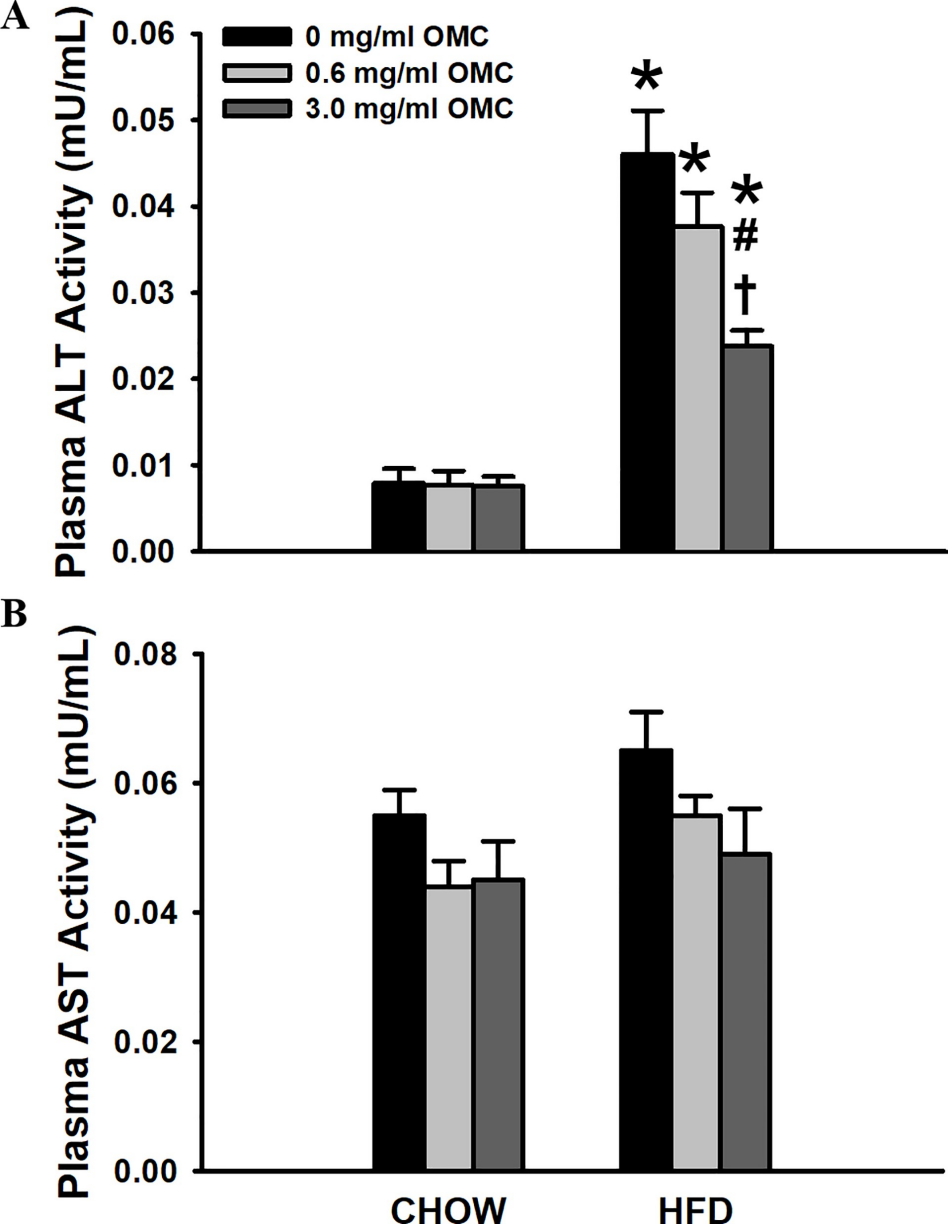
Plasma alanine aminotransferase and aspartate aminotransferase activity. Plasma ALT (A) and AST (B) activity after the 10-week diet. Data are expressed as mean ± SEM, n = 6–10 per group. Data were analyzed by two-way ANOVA. #*p*<0.05 vs. HFD control, *p<0.05 vs respective Chow, †*p*<0.05 vs HFD 0.6 mg/ml OMC.

Plasma ALT activity was significantly greater in HFD rats compared to Chow-fed animals for all doses of OMC (*p*<0.005, Fig 4A). The high dose of OMC significantly mitigated the increase in HFD-induced ALT activity compared to both the HFD control (*p*<0.001) and HFD low-dose (*p* = 0.007) treated animals (Fig 4A). Additionally, the low dose of OMC significantly reduced HFD-induced elevations in ALT activity compared to Chow-fed animals treated with the low dose (*p* < 0.001). In contrast, there were no significant differences in AST activity between or within the groups (Fig 4B).

### Endothelium-dependent vasodilation

**Fig 5:**
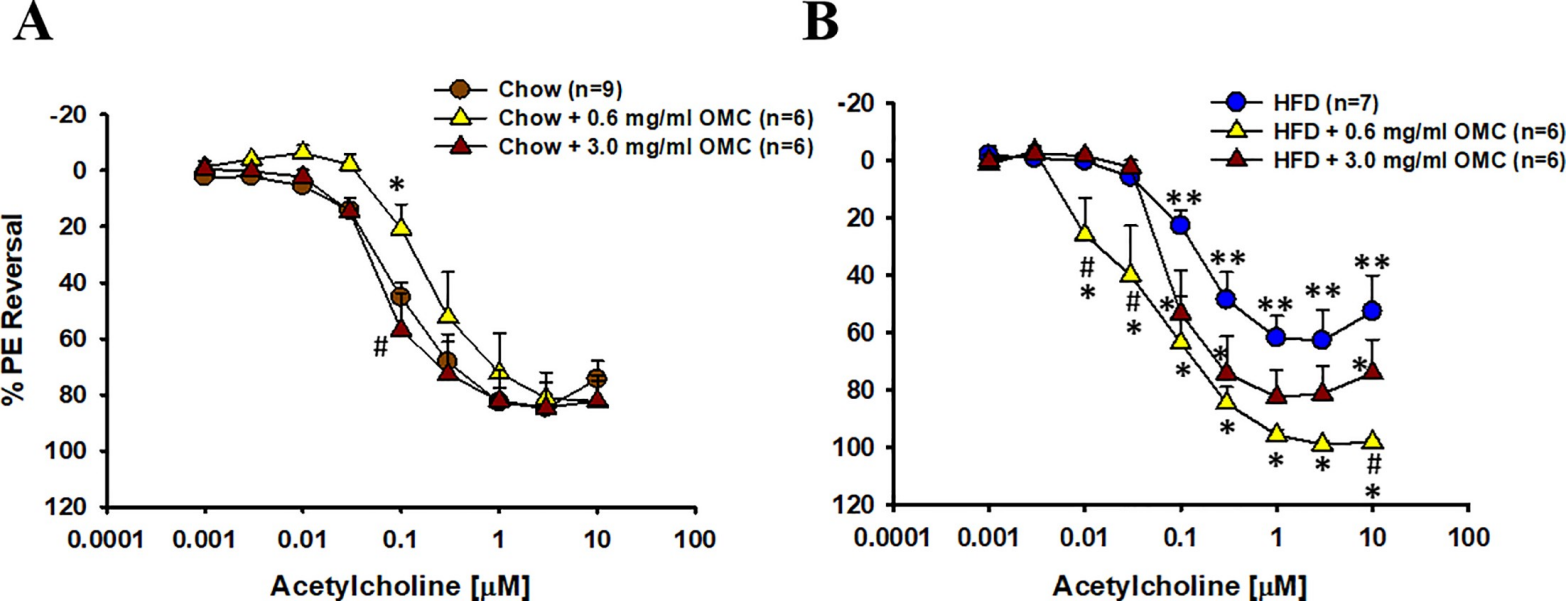
Endothelium-dependent vasodilation of arteries from Chow and HFD-fed rats treated with OMC. Isolated mesenteric resistance arteries were pre-constricted to 50% of resting inner diameter then exposed to increasing concentrations of ACh in the superfusate to obtain percent reversal of phenylephrine mediated vasodilation. Data are expressed as mean ± SEM. Data were arcsine transformed prior to analysis by two-way repeated measures ANOVA to approximate normal distribution. *p<0.05 vs respective control; **p<0.05 vs Chow control; **#**p<0.05 0.6 vs 3.0 mg/mL OMC.

Endothelium-dependent vasodilation of *ex vivo* arteries from HFD animals was significantly impaired in comparison to arteries isolated the Chow-fed controls (Fig 5). OMC was effective at both doses at preventing the HFD-induced impaired vasodilation (Fig 5B). In contrast, the low dose of OMC impaired vasodilation of *ex vivo* arteries from Chow rats, although responses to the higher doses of ACh were normal (Fig 5A). The high dose of OMC increased vasodilation compared to the low dose in arteries from Chow-fed animals (Fig 5A).

### Western blot analyses

**Fig 6:**
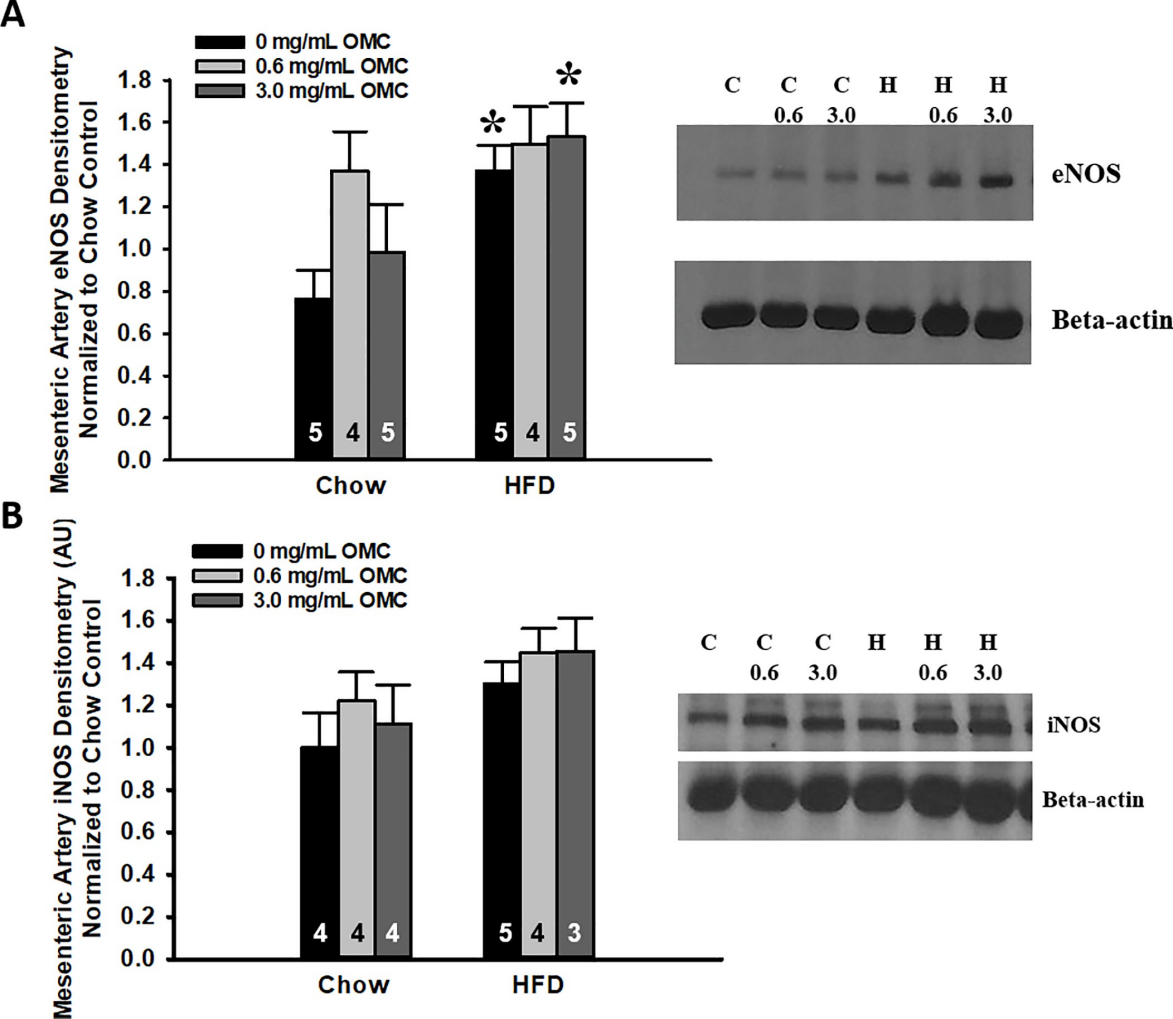
Western blot analyses of eNOS and iNOS protein expression in mesenteric arteries isolated from Chow and HFD-fed animals treated with OMC. A) Densitometry of eNOS monomer (140 kDa) protein expression. B) Densitometry of iNOS (130 kDa) protein expression. All densitometry values were normalized to the β-actin loading control and expressed as a ratio of Chow control values. Data shown as mean ± SEM. Numbers on the graphs represent sample sizes (n). *p<0.05 vs respective Chow-fed animals.

Results from the immunoblot analyses show that high fat feeding resulted in significantly greater protein expression of the inactive monomeric form of eNOS (140 kDa) (Fig 6A, Two-way ANOVA: Diet p = 0.006; OMC p = 0.122, Interaction p = 0.338). Tukey posthoc analyses show that eNOS protein expression was significantly greater in mesenteric arteries isolated from HFD rats treated with 0 mg/mL OMC (p = 0.018) and 3.0 mg/mL OMC (p = 0.031) compared to Chow-fed rats treated with the same doses of OMC. eNOS protein expression also tended to be higher in Chow-fed rats treated with 0.6 mg/mL OMC (p = 0.058). Direct comparison of Chow rats treated with 0 mg/mL and 0.6 mg/mL OMC by Student t-tests revealed a significant increase in eNOS protein expression in the OMC-treated animals (p = 0.039).

Immunoblot analysis in isolated mesenteric arteries also revealed significantly greater expression of iNOS from animals fed HFD and was unaffected by OMC treatments (Fig 6B, Two-way ANOVA: Diet p = 0.027; OMC p = 0.462; interaction p = 0.930). Tukey posthoc analyses revealed no other significant differences.

## Discussion

The present study evaluated and characterized the effect of a supplemental OMC on MetSyn-associated biochemical and pathological events in a HFD rat model. The OMC supplement is a soil-derived complex primarily consisting of a combination of minerals, trace elements, organic acids, particularly fulvic acid, nitrates and various other microbial degradation products from plant and animal origins. Results from this study demonstrated that OMC attenuated HFD-induced hyperglycemia and endothelial dysfunction, which are features of MetSyn that warrant further investigation.

Metabolic syndrome is a cluster of several risk factors that collectively are associated with increased prevalence of cardiovascular disease [19]. Diagnostically, the presence of three or more of these risk factors—abdominal obesity, elevated fasting glucose, dyslipidemias (reduced HDL and increased triglycerides), and/or hypertension–define MetSyn. Previous Metresearch from our laboratory has confirmed HFD feeding promotes symptoms consistent with MetSyn-associated sequelae [11, 12]. For example, HFD feeding to 6-week old male Sprague-Dawley rats increased body mass and abdominal adiposity, impaired endothelium-dependent vasodilation, and elevated fasting glucose. Similar findings have been reported in the Wistar rat [20, 21] thereby supporting a strain-independent, pathological similarity in this diet-induced model of MetSyn.

Protection against several MetSyn-associated pathological changes were afforded by OMC whereas others were unaffected. For example, OMC-treated animals were protected against HFD-induced hyperglycemia. This protection may be attributed to several biological or chemical properties exhibited by the materials’ primary components. First, OMC is rich in minerals and trace elements and this mineral profile may be favorable against several HFD-mediated pathophysiological endpoints including glucose and insulin regulation. In this context, increased intake of several dietary minerals have been associated with a reduced risk for developing MetSyn [22]. Additionally, fulvic acid was recently reported to stimulate insulin secretion in pigs without affecting glucose concentrations [23]. We did not observe any influence of OMC on either serum glucose or insulin levels in Chow-fed rats, but a pronounced glycemia-moderating effect was observed in HFD-treated rats. Using a very similar soil-derived mineraloid compound, leonardite, Deneau et al. [14] reported reductions in blood glucose and glycated hemoglobin employing a genetically-modified mouse model of diabetes. Mechanistically, these authors speculated some of their observed effects on glucose status were associated with increased gene expression of mitochondrial and energy-regulating enzymes. Interestingly, these researchers also reported less weight gain with their ingredient compared to control-fed animals whereas in the current study OMC had no effect on weight gain.

Overweight and obesity-central features of MetSyn-are also associated with endotoxemia, insulin resistance, hyperglycemia, and endothelial dysfunction perhaps proceeding increased generation of inflammatory cytokines and oxidative stress [24, 25]. Once initiated, oxidative stress can further impair endothelium-dependent vasodilation by increasing vascular levels of superoxide anion (O_2_·‾) and a concomitant reduction in the bioavailability of the endogenous vasodilator nitric oxide (NO) [11, 12]. Indeed, this endothelial dysfunction has been reported in obese patients [26] and was observed in *ex vivo* arteries from animals consuming HFD in the present study. The observed increases in the expression of the monomer of eNOS indicate uncoupling of eNOS, which may explain the impaired vasodilation observed following HFD and the mildly impaired vasodilation observed in the Chow animals treated with 0.6 mg/mL OMC. Similarly, the increase in iNOS protein expression in mesenteric arteries from rats fed HFD (at all doses of OMC) helps explain the impaired vasodilation that was observed as enhanced iNOS expression is frequently observed under pro-inflammatory conditions. OMC did not prevent increases in iNOS protein expression or the uncoupling of eNOS. Endogenously produced nitrates from activation of eNOS reportedly contribute up to 70% of circulating nitrites, with dietary sources of nitrates and nitrites contributing to the remainder [27].

Dietary nitrates derived from vegetables and fruits are increasingly recognized for their cardioprotective benefits [27]. These benefits are attributed to the blood pressure-lowering effects arising from conversion of dietary nitrates to nitrites by oral commensal bacteria [27]. In fact, the Dietary Approaches to Stop Hypertension (DASH) diet is thought to reduce blood pressure in part through increased consumption of fruits and vegetables resulting in dietary nitrate concentrations between 174 and 1222 mg [27]. While not comprised of fresh fruits or vegetables, analyses of OMC show the soil-derived complex contains 1230 ppm (mg/L) nitrates, which may in part explain the improved vasodilation responses in HFD fed animals treated with OMC.

Multiple factors contribute to chronic oxidative stress and inflammation in individuals with MetSyn, including chronic hyperglycemia, endotoxemia, and diets high in saturated fats. The association between HFD and saturated fat intake was described recently by Lopez-Moreno et al. [28] who noted elevated postprandial plasma LPS purportedly due to HFD promotion of LPS intestinal absorption. These endotoxins, once released from the lysis of gram-negative bacteria in the small intestine [29], induce Rac/NADPH oxidase-dependent O_2_·‾ generation by stimulating macrophage release of proinflammatory cytokines resulting in further propagation of oxidative stress [24]. Thus, despite no observed antioxidant activity by OMC, the complex significantly reduced endotoxemia. Although not measured in the current study, several potential, non-antioxidant-based mechanisms may explain this protection: 1) fulvic acid, a component of OMC, has shown anti-inflammatory activity through inhibition of ERK/JNK and COX-2 expression [30], 2) the compound also provides anti-microbial activity [31] and 3) shilajit, another earth-based complex frequently utilized in traditional medicine and rich in fulvic acid, also possesses a potent anti-ulcerative effect [32]. In addition, other multi-mineral rich natural products have demonstrated considerable hepato- and/or gastrointestinal-protection. In this context, research from Aslam et al. [33, 34] reported significant reductions in liver injury and gastrointestinal inflammation from mice fed HFD but supplemented with a mineral-rich seaweed-derived preparation. Collectively, these reports hypothesize mechanisms by which OMC may alter intestinal permeability and protect against endotoxemic damage to the gastrointestinal tract. Prior research in our laboratory has shown that urinary increases in H_2_O_2_ were not evident at 6 weeks [35]. Data from the present study show that following an additional 4 weeks of HFD resulted in increased urinary H_2_O_2_.

However, our data do not support an antioxidant role of OMC as these levels were unaffected by the supplement. It is of interest that oxidative stress was not attenuated by OMC, an observation that may suggest OMC ameliorated pathophysiological disturbances of HFD-induced metabolic disturbances via non-antioxidant-dependent mechanisms. Although fulvic acid itself has demonstrated *in vitro* antioxidant properties [36] our analysis of OMC revealed a low overall ORAC score.

Elevated levels of ROS such as H_2_O_2_ have been linked to the development of hepatic steatosis by free fatty acid peroxisomal beta-oxidation [8]. The generation of ROS can induce apoptosis of hepatocytes, promote an inflammatory response and increase ALT activity, a key indicator of liver injury [37]. Moreover, an associative relationship between liver injury and endotoxemia has been reported by others. For example, Kai et al [38] observed LPS injections exacerbated liver injury in HFD-fed rats via mechanisms implicating elevated peroxisome proliferator-activated receptors (PPARs) and beta-oxidation enzymes. Furthermore, inflammatory genes are activated in adipocytes through the generation of free radicals [39]. Our collective results of both the hepatoprotective and gastrointestinal-modifying effects of OMC observed in the current study suggest multi-faceted mechanisms contributing to its overall benefits in this model of MetSyn.

A limitation of the current study was the small sample sizes in each group, which may have limited interpretation of the outcomes. Limitations of the *ex vivo* vasodilation studies included the lack of measuring endothelium-independent responses to NO using a nitric oxide donor such as sodium nitroprusside as well as measurements of the role of oxidative stress in the vasodilatory response with each treatment. Other limitations of the current study include examination of responses in only male rodents and lack of data on blood pressure as well as food intake.

In summary, the present study demonstrated OMC, a novel and unique soil-derived product, prevented several of the complications associated with HFD-induced metabolic dysfunction. We hypothesize that the combination of organic acids, trace elements, nitrate and mineral composition of the supplement function collectively in this protection via non-antioxidant mechanisms.

## References

[1] Beltran-Sanchez H, Harhay MO, Harhay MM, McElligott S. Prevalence and trends of metabolic syndrome in the adult US population, 1999–2010. Journal of the American College of Cardiology. 2013;62:697–703. 10.1016/j.jacc.2013.05.064 23810877 PMC3756561

[2] Rochlani Y, Pothineni NV, Kovelamudi S, Mehta JL. Metabolic syndrome: Pathophysiology, management, and modulation by natural compounds. Therapeutic Advances in Cardiovascular Disease. 2017;11:215–225. 10.1177/1753944717711379 28639538 PMC5933580

[3] Bastard JP, Jardel C, Bruckert E, Blondy P, Capeau J, Laville M, et al Elevated levels of interleukin 6 are reduced in serum and subcutaneous adipose tissue of obese women after weight loss. The Journal of Clinical Endocrinology & Metabolism.2000;85:3338–3342. 10.1210/jcem.85.9.6839 10999830

[4] Tsigos C, Kyrou I, Chala E, Tsapogas P, Stavridis JC, Raptis SA, et al Circulating tumor necrosis factor alpha concentrations are higher in abdominal versus peripheral obesity. Metabolism. 1999;48:1332–1335. 10.1016/S0026-0495(99(90277-9 10535400

[5] Almeda-Valdes P, Herrera-Mercadillo RJ, Aguilar-Salinas CA, Uribe M, Méndez-Sánchez N. The role of diet in patients with metabolic syndrome. Current Medicinal Chemistry. 2017. 10.2174/0929867324666170518095316 28521684

[6] Ostman C, Smart NA, Morcos D, Duller A, Ridley W, Jewiss D. The effect of exercise training on clinical outcomes in patients with the metabolic syndrome: a systematic review and meta-analysis. Cardiovascular Diabetology. 2017;16:110 10.1186/st12933-017-0590-y 28854979 PMC5577843

[7] Azadbakht L, Mirmiran P, Esmaillzadeh A, Azizi T, Azizi F. Beneficial effects of a Dietary Approaches to Stop Hypertension eating plan on features of the metabolic syndrome. Diabetes Care. 2005;28:2823–2831. 10.2337/diacare.28.12.2823 16306540

[8] Gusdon AM, Song K, Qu S. Nonalcoholic fatty liver disease: pathogenesis and therapeutics from a mitochondria-centric perspective. Oxidative Medicine and Cellular Longevity. 2014;637027 10.1155/2014/637027 25371775 PMC4211163

[9] Moss JWE, Williams JO, Ramji DP. Nutraceuticals as therapeutic agents for atherosclerosis. Biochimica et Biophysica Acta. 2018;1864:1562–1572. 10.1016/j.bbadis.2018.02.006 29454074 PMC5906642

[10] Ríos-Hoyo A, Cortés MJ, Ríos-Ontiveros H, Meaney E, Ceballos G, Gutiérrez-Salmeán G. Obesity, metabolic syndrome, and dietary therapeutical approaches with a special focus on nutraceuticals (polyphenols): a mini-review. International Journal of Vitamin and Nutrition Research. 2014;84:113–123. 10.1024/0300-9831/a000198 26098475

[11] Sweazea KL, Lekic M, Walker BR. Comparison of mechanisms involved in impaired vascular reactivity between high sucrose and high fat diets in rats. Nutrition and Metabolism (Lond). 2010;7:48 10.1186/1743-7075-7-48 PMC2887873 20525365

[12] Sweazea KL, Walker BR. High fat feeding impairs endothelin-1 mediated vasodilation through increased iNOS-derived nitric oxide. Hormone and Metabolic Research. 2011;43:470–476. 10.1055/s-0031-1273763 21448844 PMC3376914

[13] Crawford M, Whisner C, Al-Nakkash L, Sweazea KL. Six-week high fat diet alters the gut microbiome and promotes cecal inflammation, endotoxin production and simple steatosis without obesity in male rats. Lipids. 2019;54:119–131. 10.1002/lipd.12131 30860608

[14] Deneau J, Ahmed T, Blotsky R, Bojanowski K. Anti-diabetic activity of a mineraloid isolate, in vitro and in genetically diabetic mice. International Journal of Vitamin and Nutrition Research. 2011;81:34–42. 10.1024/0300-9831/a000048 22002216

[15] Bernardis LL. Prediction of carcass fat, water and lean body mass from Lee’s “nutritive ratio” in rats with hypothalamic obesity. Experientia. 1970;26:789–790. 10.1007/BF02232553 4914444

[16] Novelli EL, Diniz YS, Galhardi CM, Ebaid GM, Rodrigues HG, Mani F, et al Anthropometrical parameters and markers of obesity in rats. Laboratory Animal. 2007;41,111–119. 10.1258/002367707779399518 17234057

[17] Bowe JE, Franklin ZJ, Hauge-Evans AC, King AJ, Persaud SJ, Jones PM. Metabolic phenotyping guidelines: assessing glucose homeostasis in rodent models. Journal of Endocrinology. 2014;222(3):G13–G25. 10.1530/JOE-14-0182 25056117

[18] Chen H, Sullivan G, Quon MJ. Assessing the predictive accuracy of QUICKI as a surrogate index for insulin sensitivity using a calibration model. Diabetes. 2005;54(7):1914–1925. 10.2337/diabetes.54.7.1914 15983190

[19] Alberti KG, Eckel RH, Grundy SM, Zimmet PZ, Cleeman JI, Donato KA, et al Harmonizing the metabolic syndrome: a joint interim statement of the International Diabetes Federation Task Force on Epidemiology and Prevention; National Heart, Lung, and Blood Institute; American Heart Association; World Heart Federation; International Atherosclerosis Society; and International Association for the Study of Obesity. Circulation. 2009;120:1640–1645. 10.1161/CIRCULATIONAHA.109.192644 19805654

[20] Auberval N, Dal S, Bietiger W, Pinget M, Jeandidier N, Maillard-Pedracini E, et al Metabolic and oxidative stress markers in Wistar rats after 2 months on a high-fat diet. Diabetology & Metabolic Syndrome. 2014;6:130 10.1186/1758-5996-6-130 25960774 PMC4424531

[21] Panchal SK, Poudyal H, Iyer A, Nazer R, Alam MA, Diwan V, et al High-carbohydrate, high-fat diet-induced metabolic syndrome and cardiovascular remodeling in rats. Journal of Cardiovascular Pharmacology. 2012;57:611–624. 10.1097/FJC.0b013e3181feb90a 21572266

[22] Robberecht H, De Bruyne T, Hermans N. Biomarkers of the metabolic syndrome: Influence of minerals, oligo- and trace elements. Journal of Trace Elements in Medicine and Biology. 2017;43:23–28. 10.1016/j.jtemb.2016.10.005 28277234

[23] Chang Q, Lu Z, He M, Gao R, Bai H, Shi B, et al Effects of dietary supplementation of fulvic acid on lipid metabolism of finishing pigs. Journal of Animal Science. 2014;92:4921–4926. 10.2527/jas/2014-8137 25349342

[24] Hsu H-Y, Wen M-H. Lipopolysaccharide-mediated reactive oxygen species and signal transduction in the regulation of interleukin-1 gene expression. Journal of Biological Chemistry. 2002;277:22131–22139. 10.1074/jbc.M111883200 11940570

[25] McMurray F, Patten DA, Harper ME. Reactive oxygen species and oxidative stress in obesity—recent findings and empirical approaches. Obesity. 2016;24:2301–2310. 10.1002/oby.21654 27804267

[26] Van Guilder GP, Hoetzer GL, Dengel DR, Stauffer BL, DeSouza CA. Impaired endothelium-dependent vasodilation in normotensive and normoglycemic obese adult humans. Journal of Cardiovascular Pharmacology. 2006;47:310–313. 10.1097/01.fjc0000205097.29946.d3 16495771

[27] Hord NG, Tang Y, Bryan NS. Food sources of nitrates and nitrites: The physiologic context for potential health benefits. The American Journal of Clinical Nutrition. 2009;90(1):1–10. 10.3945/ajcn.2008.27131 19439460

[28] Lopez-Moreno J, Garcia-Carpintero S, Jimenez-Lucena R, Haro C, Rangel-Zuniga OA, Blanco-Rojo R, et al Effect of dietary lipids on endotoxemia influences postprandial inflammatory response. Journal of Agriculture and Food Chemistry. 2017;65:7756–7763. 10.1021/acs.jafc.7b01909 28793772

[29] Wang X, Quinn P. Endotoxins: Lipopolysaccharides of Gram-Negative Bacteria. Subcellular Biochemistry. 2010;53:3–25. 10.1007/978-90-481-9078-2 20593260

[30] Chien SJ, Chen TC, Kuo HC, Chen CN, Chang SF. Fulvic acid attenuates homocysteine-induced cyclooxygenase-2 expression in human monocytes. BMC Complementary and Alternative Medicine. 2015;15:61 10.1186/s12905-015-0583-x 25888188 PMC4369892

[31] Van Rensburg CE, van Straten A, Dekker J. An *in vitro* investigation of the antimicrobial activity of oxifulvic acid. Journal of Antiomicrobial Chemotherapy. 2000;46:853 doi: 10/1093/jac/46.5.853 10.1093/jac/46.5.853 11062218

[32] Ghosal S, Singh SK, Kumar Y, Srivastava R, Goel RK, Dey R, et al Anti-ulcerogenic activity of fulvic acids and 4’-methoxy-6-carbomethoxybiphenyl isolated from shilajit. Phytotherapy Research. 1988;2:187–191. 10.1002/ptr.2650020408

[33] Aslam MN, Paruchuri T, Bhagavathula N, Varani J. A mineral-rich red algae extract inhibits polyp formation and inflammation in the gastrointestinal tract of mice on a high-fat diet. Integrative Cancer Therapies. 2010;9:93–99. 10.1177/1534735409360360 20150219 PMC2861409

[34] Aslam MN, Bergin I, Naik M, Paruchuri T, Hampton A, Rehman, et al A multi-mineral natural product inhibits liver tumor formation in C57BL/6 mice. Biological Trace Element Research. 2012;147:267–274. 10.1080/01635581.2012.713160 22222483 PMC3360994

[35] Crinigan C, Calhoun M, Sweazea KL. Short-term high fat intake does not significantly alter markers of renal function or inflammation in young male Sprague-Dawley rats. Journal of Nutrition and Metabolism. 2015;157520 10.1155/2015/157520 26185688 PMC4491386

[36] Rodriguez NC, Urrutia EC, Gertrudis BH, Chaverri JP, Mejia GB. Antioxidant activity of fulvic acid: a living matter-derived bioactive compound. Journal of Food, Agriculture & Environment. 2011;9:123–127.

[37] Li S, Tan H-Y, Wang N, Zhang ZJ, Lao L, Wong CW, et al The role of oxidative stress and antioxidants in liver diseases. International Journal of Molecular Sciences. 2015;16:26087–26124. 10.3390/ijms161125942 26540040 PMC4661801

[38] Kai M, Miyoshi M, Fujiwara M, Nishiyama Y, Inoue T, Maeshige N, et al A lard-rich high-fat diet increases hepatic peroxisome proliferator-activated receptors in endotoxemic rats. Journal of Surgical Research. 2017;212:22–32. 10.1016/j.jss.2016.11.048 28550910

[39] Han CY. Roles of reactive oxygen species on insulin resistance in adipose tissue. Diabetes & Metabolism Journal. 2016;40:272–279. 10.4093/dmj.2016.40.4.272 27352152 PMC4995181

[40] Lamar RT, Olk DC, Mayhew L, Bloom PR. A new standardized method for quantification of humic and fulvic acids in humic ores and commercial product. Journal of AOAC International. 2014;97:721–730. 10.5740/jaoacint.13-393 25051616

